# Supplementary material for: Amplified DNA heterogeneity assessment with Oxford Nanopore sequencing applied to cell free expression templates
Source: PLoS One. 2024 Dec 3;19(12):e0305457. doi: 10.1371/journal.pone.0305457 (PMC11614277; doi:10.1371/journal.pone.0305457)
Supplement: S1 File — (DOCX) [file pone.0305457.s001.docx]

**Supplement 1**

**Supplement S1 Text**: Linear Expression Templates used in this study

**Supplement Table S1**- results of the De novo assembly of the short read NGS sequences to make larger contigs

**Supplement Table S2**- differences between expected results and observed results of Illumina sequencing for frGFP and sfGFP mixtures

**Supplement Table S3**- differences between expected results and observed results of Illumina sequencing for frGFP and sfGFP mixtures

**Supplement Table S4**- Statistical analysis of significance on error-introduced samples of sfGFP and frGFP

**Supplement Table S5**- Statistical analysis of significance of amplified samples.

**Supplement Table S6**- Cigar File abbreviation list.

**Supplement Figure S1** distribution of Q-scores for the sequenced read of different Nanopore samples

**Supplement Figure S2:** coverage of mapped illumina sequenced of different errored samples to the sfGFP reference genome.

**Supplement Figure S3**: distribution of Error rates for different mixtures of frGFP and sfGFP sequenced by Illumina sequencing

**Supplement Figure S4**: distribution of Error rates for different mixtures of frGFP and sfGFP sequenced by Illumina sequencing.

**Supplement Figure S5:**  CIGAR string dividing and part explanation

**Supplement Figure S6:** Length distribution of ONT nanopore reads of different PCR amplification methods.

**Supplement Code S1** – Snakefile summarizing all of the steps of DNA data analysis.

**Supplement Code S2:** error analysis data extraction program for Nanopore data analysis.

**Supplement code S3** : a python program written to map the error’s location on reference sequence

**Supplement code S4** : a python program written to fit Lorentzian function to emission data of sfGFP and measures FWHM of the peak.

## Linear Expression Templates

Below are the genetic templates used in sequencing and CFE. All sequences were optimized for *Escherichia coli* using IDT’s codon optimization tool. Each color represent the region according to Addgene pJl1-sfGFP gene for the Plasmid vector and our added sites for making circularized templates:

T7 Promoter – T7 Promoter

RBS – Ribosome binding site

Start – Start codon

Protein Sequence – Gene coding for protein

Stop – Stop codon

T7 Terminator – T7 Terminator

Circularization site – HindIII Digest

Primer Sequences – Primer sequences

Ori – Ori

KanR – KanR

sfGFP LET – 999 bp

GTAAAACGACGGCCAGTAGCGCTATTAAAGCTTcgaaatTAATACGACTCACTATAGGGAGACCACAACGGTTTCCCTCTAGAAATAATTTTGTTTAACTTTAAGAAGGAGATATACATATGAGCAAAGGTGAAGAACTGTTTACCGGCGTTGTGCCGATTCTGGTGGAACTGGATGGCGATGTGAACGGTCACAAATTCAGCGTGCGTGGTGAAGGTGAAGGCGATGCCACGATTGGCAAACTGACGCTGAAATTTATCTGCACCACCGGCAAACTGCCGGTGCCGTGGCCGACGCTGGTGACCACCCTGACCTATGGCGTTCAGTGTTTTAGTCGCTATCCGGATCACATGAAACGTCACGATTTCTTTAAATCTGCAATGCCGGAAGGCTATGTGCAGGAACGTACGATTAGCTTTAAAGATGATGGCAAATATAAAACGCGCGCCGTTGTGAAATTTGAAGGCGATACCCTGGTGAACCGCATTGAACTGAAAGGCACGGATTTTAAAGAAGATGGCAATATCCTGGGCCATAAACTGGAATACAACTTTAATAGCCATAATGTTTATATTACGGCGGATAAACAGAAAAATGGCATCAAAGCGAATTTTACCGTTCGCCATAACGTTGAAGATGGCAGTGTGCAGCTGGCAGATCATTATCAGCAGAATACCCCGATTGGTGATGGTCCGGTGCTGCTGCCGGATAATCATTATCTGAGCACGCAGACCGTTCTGTCTAAAGATCCGAACGAAAAACGGGACCACATGGTTCTGCACGAATATGTGAATGCGGCAGGTATTACGTGGAGCCATCCGCAGTTCGAAAAATAATAAGTCGACCGGCTGCTAACAAAGCCCGAAAGGAAGCTGAGTTGGCTGCTGCCACCGCTGAGCAATAACTAGCATAACCCCTTGGGGCCTCTAAACGGGTCTTGAGGGGTTTTTTGCTGAAAGCGAGACTAAGCTTTAAACTTCGGGTCATAGCTGTTTCCTG

pJL1-sfGFP – 2486 bp (reverse complementary)

GATGCCACGATTGGCAAACTGACGCTGAAATTTATCTGCACCACCGGCAAACTGCCGGTGCCGTGGCCGACGCTGGTGACCACCCTGACCTATGGCGTTCAGTGTTTTAGTCGCTATCCGGATCACATGAAACGTCACGATTTCTTTAAATCTGCAATGCCGGAAGGCTATGTGCAGGAACGTACGATTAGCTTTAAAGATGATGGCAAATATAAAACGCGCGCCGTTGTGAAATTTGAAGGCGATACCCTGGTGAACCGCATTGAACTGAAAGGCACGGATTTTAAAGAAGATGGCAATATCCTGGGCCATAAACTGGAATACAACTTTAATAGCCATAATGTTTATATTACGGCGGATAAACAGAAAAATGGCATCAAAGCGAATTTTACCGTTCGCCATAACGTTGAAGATGGCAGTGTGCAGCTGGCAGATCATTATCAGCAGAATACCCCGATTGGTGATGGTCCGGTGCTGCTGCCGGATAATCATTATCTGAGCACGCAGACCGTTCTGTCTAAAGATCCGAACGAAAAAGGCACGCGGGACCACATGGTTCTGCACGAATATGTGAATGCGGCAGGTATTACGTGGAGCCATCCGCAGTTCGAAAAATAAGTCGACCGGCTGCTAACAAAGCCCGAAAGGAAGCTGAGTTGGCTGCTGCCACCGCTGAGCAATAACTAGCATAACCCCTTGGGGCCTCTAAACGGGTCTTGAGGGGTTTTTTGCTGAAAGCCAATTCTGATTAGAAAAACTCATCGAGCATCAAATGAAACTGCAATTTATTCATATCAGGATTATCAATACCATATTTTTGAAAAAGCCGTTTCTGTAATGAAGGAGAAAACTCACCGAGGCAGTTCCATAGGATGGCAAGATCCTGGTATCGGTCTGCGATTCCGACTCGTCCAACATCAATACAACCTATTAATTTCCCCTCGTCAAAAATAAGGTTATCAAGTGAGAAATCACCATGAGTGACGACTGAATCCGGTGAGAATGGCAAAAGCTTATGCATTTCTTTCCAGACTTGTTCAACAGGCCAGCCATTACGCTCGTCATCAAAATCACTCGCATCAACCAAACCGTTATTCATTCGTGATTGCGCCTGAGCGAGACGAAATACGCGATCGCTGTTAAAAGGACAATTACAAACAGGAATCGAATGCAACCGGCGCAGGAACACTGCCAGCGCATCAACAATATTTTCACCTGAATCAGGATATTCTTCTAATACCTGGAATGCTGTTTTCCCGGGGATCGCAGTGGTGAGTAACCATGCATCATCAGGAGTACGGATAAAATGCTTGATGGTCGGAAGAGGCATAAATTCCGTCAGCCAGTTTAGTCTGACCATCTCATCTGTAACATCATTGGCAACGCTACCTTTGCCATGTTTCAGAAACAACTCTGGCGCATCGGGCTTCCCATACAATCGATAGATTGTCGCACCTGATTGCCCGACATTATCGCGAGCCCATTTATACCCATATAAATCAGCATCCATGTTGGAATTTAATCGCGGCTTCGAGCAAGACGTTTCCCGTTGAATATGGCTCATAACACCCCTTGTATTACTGTTTATGTAAGCAGACAGTTTTATTGTTCATGATGATATATTTTTATCTTGTGCAATGTAACATCAGAGATTTTGAGACACAACGTGAGATCAAAGGATCTTCTTGAGATCCTTTTTTTCTGCGCGTAATCTGCTGCTTGCAAACAAAAAAACCACCGCTACCAGCGGTGGTTTGTTTGCCGGATCAAGAGCTACCAACTCTTTTTCCGAAGGTAACTGGCTTCAGCAGAGCGCAGATACCAAATACTGTTCTTCTAGTGTAGCCGTAGTTAGGCCACCACTTCAAGAACTCTGTAGCACCGCCTACATACCTCGCTCTGCTAATCCTGTTACCAGTGGCTGCTGCCAGTGGCGATAAGTCGTGTCTTACCGGGTTGGACTCAAGACGATAGTTACCGGATAAGGCGCAGCGGTCGGGCTGAACGGGGGGTTCGTGCACACAGCCCAGCTTGGAGCGAACGACCTACACCGAACTGAGATACCTACAGCGTGAGCTATGAGAAAGCGCCACGCTTCCCGAAGGGAGAAAGGCGGACAGGTATCCGGTAAGCGGCAGGGTCGGAACAGGAGAGCGCACGAGGGAGCTTCCAGGGGGAAACGCCTGGTATCTTTATAGTCCTGTCGGGTTTCGCCACCTCTGACTTGAGCGTCGATTTTTGTGATGCTCGTCAGGGGGGCGGAGCCTATGGAAAAACGCCAGCAACGCGATCCCGCGAAATTAATACGACTCACTATAGGGAGACCACAACGGTTTCCCTCTAGAAATAATTTTGTTTAACTTTAAGAAGGAGATATACATATGAGCAAAGGTGAAGAACTGTTTACCGGCGTTGTGCCGATTCTGGTGGAACTGGATGGCGATGTGAACGGTCACAAATTCAGCGTGCGTGGTGAAGGTGAAGGC

frGFP (errors introduced to adjust mutations)

GTAAAACGACGGCCAGTAGGCTATTAAAGCTTCGAAATTTAATACGACTCACTATAGGGAGACCACAACGGTTCCCTCTAAGAAATAATTTTGTTTAACTTTAAGAAGGAGATATACATATGAGCAAAGGTGAAGAACTGTTTACCGGCGTTGTGCCGATTCTGGTGGAACTGGATGGCGATGTGAACGGTCACAAATTCAGCGTGAGCGGTGAAGGTGAAGGCGATGCCACGTACGGCAAACTGACGCTGAAATTTATCTGCACCACCGGCAAACTGCCGGTGCCGTGGCCGACGCTGGTGACCACCCTGACCTATGGCGTTCAGTGTTTTAGTCGCTATCCGGATCACATGAAACGTCACGATTTCTTTAAATCTGCAATGCCGGAAGGCTATGTGCAGGAACGTACGATTAGCTTTAAAGATGATGGCAATTATAAAACGCGCGCCGAGGTGAAATTTGAAGGCGATACCCTGGTGAACCGCATTGAACTGAAAGGCATAGATTTTAAAGAAGATGGCAATATCCTGGGCCATAAACTGGAATACAACTATAATAGCCATAATGTTTATATTACGGCGGATAAACAGAAAAATGGCATCAAAGCGAATTTTAAAATTCGCCATAACATAGAAGATGGCAGTGTGCAGCTGGCAGATCATTATCAGCAGAATACCCCGATTGGTGATGGTCCGGTGCTGCTGCCGGATAATCATTATCTGAGCACGCAGTCGGCACTGTCTAAAGATCCGAACGAAAAACGTGACCACATGGTATTATTAGAATTTGTAACTGCCGCTGGAATCACTCACGGTATGGACGAATTATACAAATAATAAGTCGACGGCTGCTAACAAAGCCCGAAAGGAAGCTGAGTTGGCTGCTGCCACCGCTGAGCAATAACTAGCATAACCCCTTGGGGCCTCTAAACGGGTCTTGAGGGGTTTTTTGCTGAAAGCGAGAACTAAGCTTTAAACTTCGGGTCATAGCTGTTTCCTG

## Quality scores for different samples


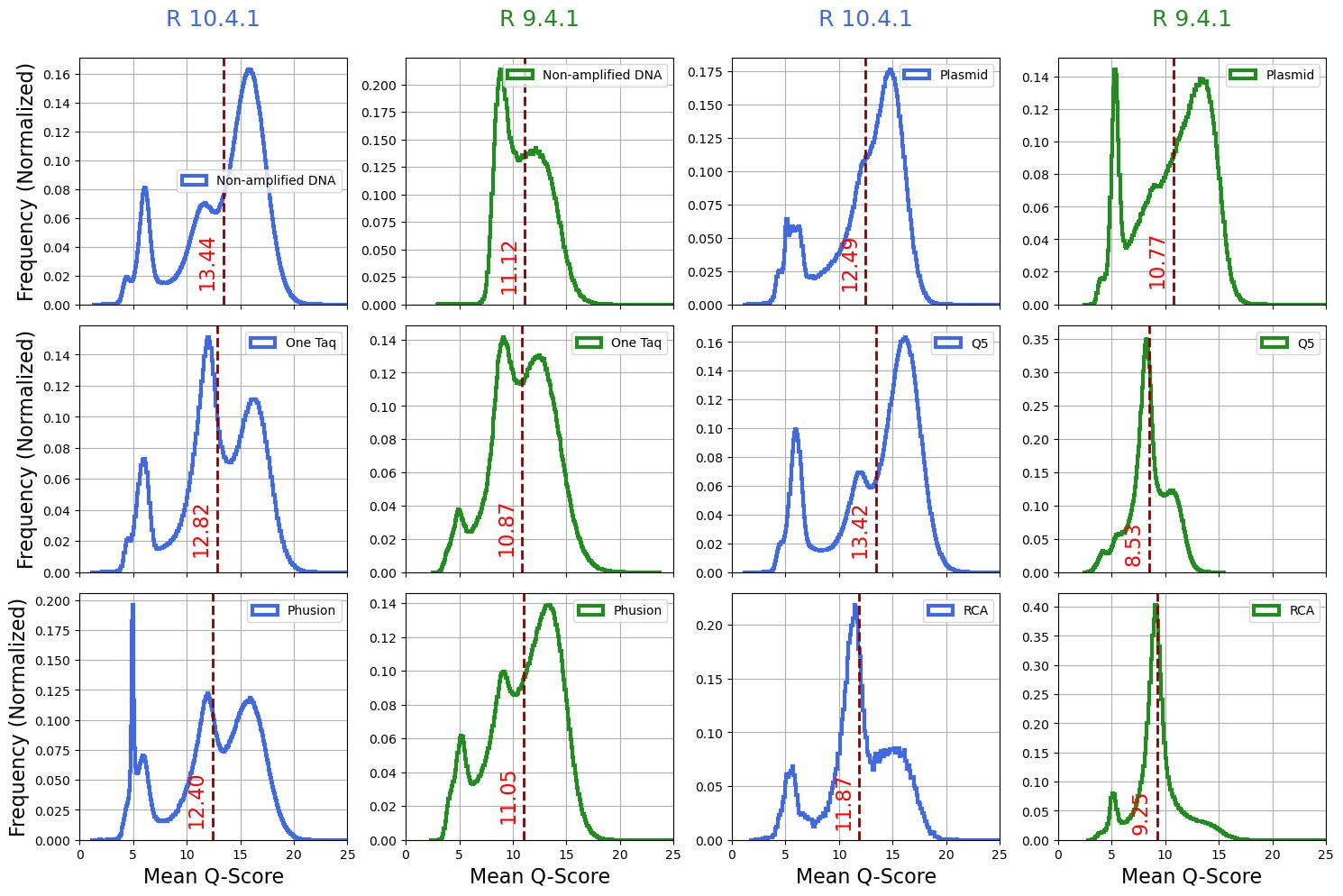


Figure S1 distribution of Q-scores for the sequenced read of different Nanopore samples. The y-axis is the probability density and the X-Axis is the Mean Q-score. The red-dashed lines are the averages of each plot.

## NGS vs nanopore data

Table S1- results of the De novo assembly of the short read NGS sequences to make larger contigs

| **Sample** | **Reads** | **Contigs** | **N75 (bp)** | **N50(bp)** | **N25(bp)** | **Minimum(bp)** | **Maximum(bp)** | **Average(bp)** |
| --- | --- | --- | --- | --- | --- | --- | --- | --- |
|  |  |  |  |  |  |  |  |  |
| **sfGFP** | 187,530 | 32 | 349 | 406 | 497 | 207 | 706 | 402 |
| **mixed 1:1000** | 171,346 | 32 | 345 | 395 | 451 | 244 | 534 | 379 |
| **mixed 1:100** | 160,872 | 46 | 370 | 402 | 459 | 259 | 580 | 404 |
| **mixed 1:10** | 136,810 | 8 | 424 | 474 | 484 | 281 | 622 | 444 |
| **frGFP** | 261,082 | 27 | 320 | 379 | 488 | 150 | 734 | 362 |

**Table S2**- differences between expected results and observed results of Illumina sequencing for frGFP and sfGFP mixtures

| **error type** | **mixture** | **difference from expected (%)** |
| --- | --- | --- |
| Substitution | 1:1000 mix | 8.709 |
| Substitution | 1:100 mix | -14.211 |
| Substitution | 1:10 mix | 28.408 |
| Deletion | 1:1000 mix | 1.112 |
| Deletion | 1:100 mix | 0.140 |
| Deletion | 1:10 mix | 9.059 |
| Insertion | 1:1000 mix | 12.819 |
| Insertion | 1:100 mix | -12.236 |
| Insertion | 1:10 mix | 33.334 |

**Table S3**- differences between expected results and observed results of Illumina sequencing for frGFP and sfGFP mixtures

| **error type** | **mixture** | **difference from expected (%)** |
| --- | --- | --- |
| Substitution | 1:1000 mix | 13.958 |
| Substitution | 1:100 mix | 11.314 |
| Substitution | 1:10 mix | 5.351 |
| Deletion | 1:1000 mix | 1.216 |
| Deletion | 1:100 mix | 2.587 |
| Deletion | 1:10 mix | 4.058 |
| Insertion | 1:1000 mix | 6.791 |
| Insertion | 1:100 mix | 10.533 |
| Insertion | 1:10 mix | 10.217 |

## Error rate distributions and coverage

## Coverage of Illumina sequenced data on the reference sfGFP


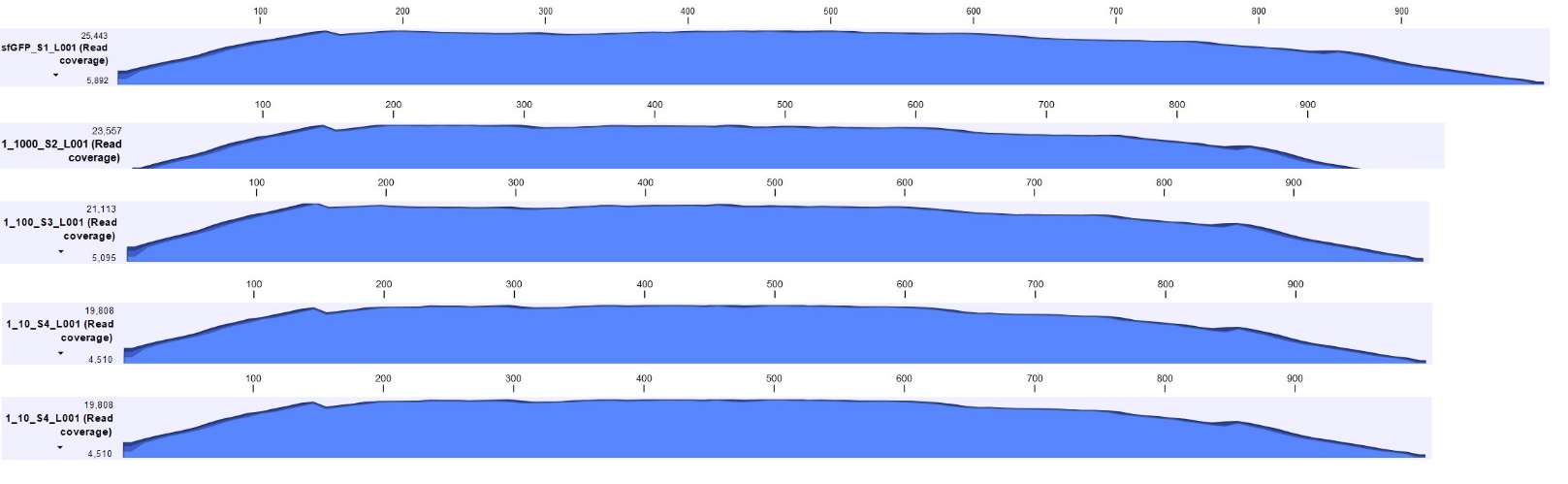


**Figure S2** – coverage of mapped illumina sequenced of different errored samples to the sfGFP reference genome.

### Illumina data error distribution for error-introduced samples


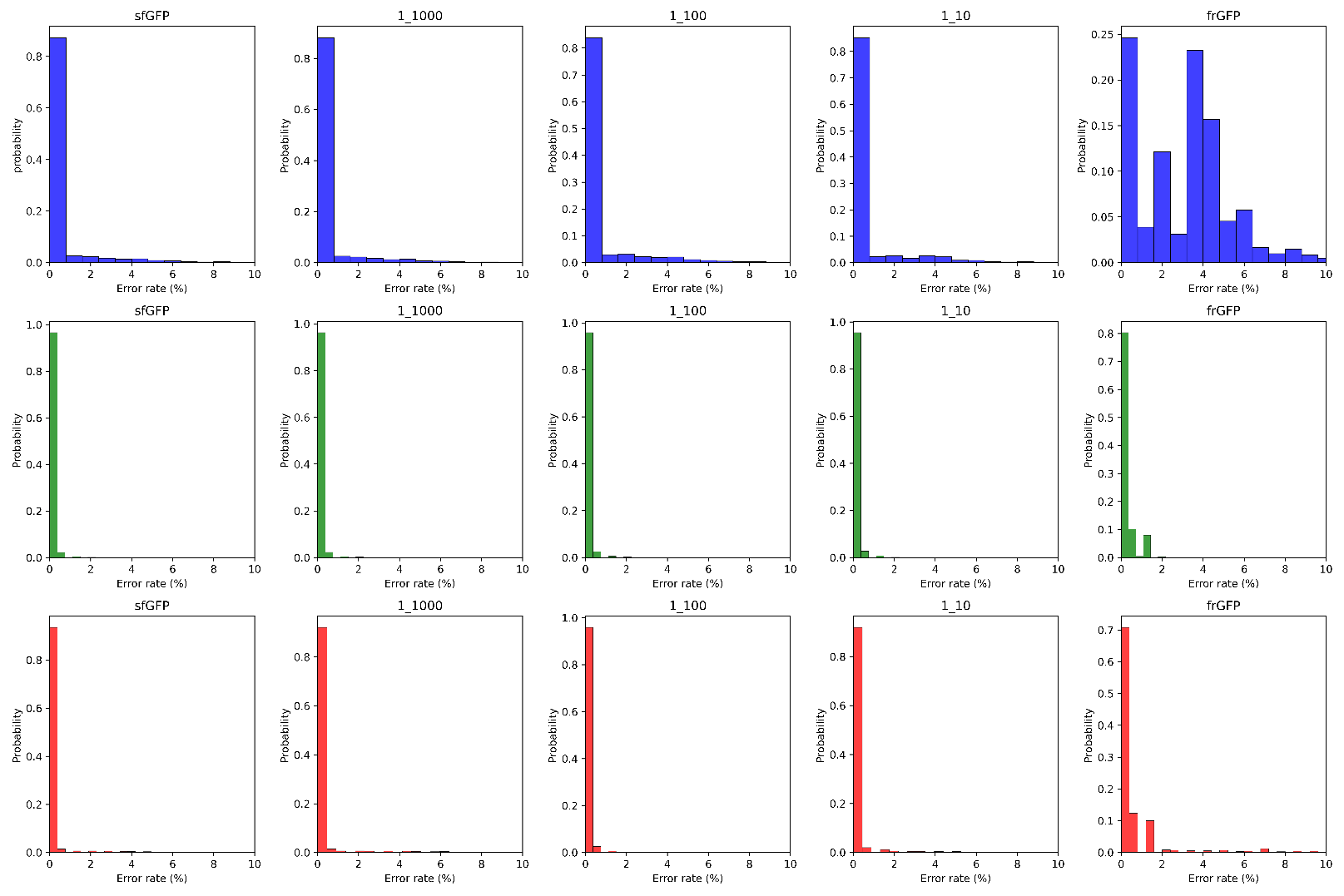


**Figure S3** distribution of Error rates for different mixtures of frGFP and sfGFP sequenced by Illumina sequencing. The Y axis presents the probability of each error histogram bin and the X axis is the error rate (%).

### ONT data error distribution for error-introduced samples





Figure S4 distribution of Error rates for different mixtures of frGFP and sfGFP sequenced by Illumina sequencing. The Y axis presents the probability of each error histogram bin and the X axis is the error rate (%).

## Statistical significance analysis for distributions

All of the samples were analyzed by bootstrapping approach and tested which select multiple samples with specified sizes from two data, and a Mann-Whitney U test was applied to the samples. By iterations, an average of p-values of the Mann-Whitney u test is reported as the p-value for two distributions compared.

**Statistical analysis for error introduced samples**

Table S4- Statistical analysis of significance on error-introduced samples of sfGFP and frGFP

### Statistical analysis amplified samples

Table S5- Statistical analysis of significance of amplified samples

## CIGAR string example

This section contains an explanatory figure for CIGAR string analysis.


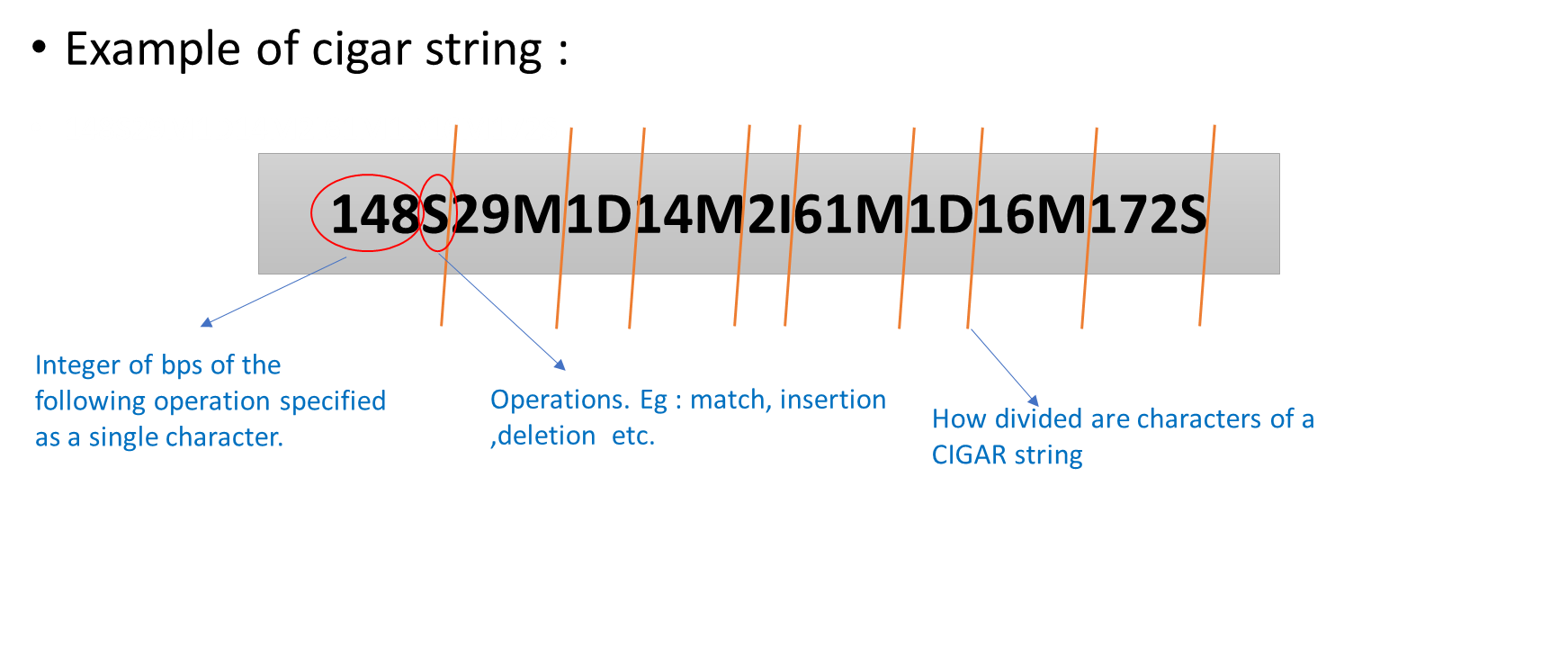


**Figure S5** – CIGAR string dividing and part explanation

Table S6- Cigar File abbreviation list

| Operation | description |
| --- | --- |
| M | Match (alignment column containing two letters). This could contain two different letters (mismatch) X or two identical letters = depending on the code used for mapping. |
| D | Deletion (gap in the target sequence). |
| I | Insertion (gap in the query sequence). |
| S | Segment of the query sequence that does not appear in the alignment. This is used with soft clipping, where the full-length query sequence is given (field 10 in the SAM record). In this case, S operations specify segments at the start and/or end of the query that do not appear in a local alignment. |
| H | \| Segment of the query sequence that does not appear in the alignment. This is used with hard clipping, where only the aligned segment of the query sequences is given (field 10 in the SAM record). In this case, H operations specify segments at the start and/or end of the query that do not appear in the SAM record. \| \| --- \| |
| = | Alignment column containing two identical letters. USEARCH can read CIGAR strings using this operation, but does not generate them. |
| X | Alignment column containing a mismatch, i.e. two different letters. USEARCH can read CIGAR strings using this operation, but does not generate them. |

## computational data

Data sequencing was performed and saved in the FAST5 format. The guppy_base caller version 4.5.3+0ab5ebbc3, along with the NVIDIA GeForce RTX 3090, NVIDIA driver version 460.91.03, and CUDA version 11.2 on Ubuntu 20.04 LTS were utilized to basecall all FAST5 files. Subsequently, the FASTQ files obtained from the basecalling process were merged without considering their Q score, and employed for further analysis of the data. The resulting sam files were sorted, indexed, and converted to the bam format using Samtools. The sorted bam files were then converted to csv format to simplify the downstream import and analysis as a Python Pandas DataFrame using Snakefile code.

Data analysis were performed by analyzing the CIGAR string to extract information about deletion, insertion and identity(substitution) errors using codes 2,3.

**Supplement Code S1** – Snakefile summarizing all of the steps of DNA data analysis.

"""

Snakemake pipeline for Nanopore data analysis

Code developed by Denis Tamiev

"""

import glob

import os

import glob

from Bio import SeqIO

### Setting Parameters for this Run ###

curDir=str(os.getcwd()) #dir where the snakefile is located

curDir=str(curDir + "/")

#auto detect the name of the ref genomes in <./ref/*.fasta> file

i=0

for seq_record in SeqIO.parse(str(curDir + "ref/fullConstruct.fasta"), "fasta"):

if i==0: #first reference sequence is plasmid

setPlasmidName =seq_record.id

if i==1: #second reference sequence is genome

setRefGenomeName=seq_record.id

i+=1

print("#####################################################################")

print("GENOME NAME:", setRefGenomeName)

print("PLASMID NAME:", setPlasmidName)

print("CURRENT DIRECTORY:", curDir)

print("#####################################################################")

### A list of Rules ###

## 1

# Merges all fastq files

rule createMergedFQ:

input:

fqFail=glob.glob("tempData/fail/*.fastq"),

fqPass=glob.glob("tempData/pass/*.fastq")

output:

mergedTxtPartial="tempData/merged_all.txt"

run:

shell("cat {input.fqFail} | tee -a {output.mergedTxtPartial}") # Adds all fqFail files to the merged_all.txt

shell("cat {input.fqPass} | tee -a {output.mergedTxtPartial}") # Adds all fqPass files to the merged_all.txt

## 2

# Converts all fastq from txt to fastq

rule moveFQ:

input:

fqAll="tempData/merged_all.txt"

output:

fqDest="data/fastq/merged_all.fastq"

shell:

"mv {input.fqAll} {output.fqDest}"

## 3

# Aligns fastq to reference genome

rule minimap2_align:

input:

fa="ref/fullConstruct.fasta",

fq="data/fastq/merged_all.fastq"

output:

samFile="alignment/samfile.sam"

shell:

"minimap2 -ax map-ont {input.fa} {input.fq} > {output}"

## 4

#convert EACH .sam to .bam

rule sam_to_bam:

input:

"alignment/samfile.sam"

output:

"alignment/merged.bam"

shell:

"samtools view -b {input} > {output}"

## 5

#sort merged.bam

rule samtools_sort:

input:

"alignment/merged.bam"

output:

"alignment/merged_sorted.bam"

shell:

"samtools sort -T alignment/merged "

"-O bam {input} > {output}"

## 6

#index the sorted alignment file

rule samtools_index:

input:

"alignment/merged_sorted.bam"

output:

"alignment/merged_sorted.bam.bai"

shell:

"samtools index {input}"

## 7

# Convert .bam files to .csv

bamDir=str(curDir + "alignment/merged_sorted.bam")

rule convertToCSV:

input:

bamId = "alignment/merged_sorted.bam.bai",

params:

prefix=bamDir

output:

"scripts/bamToDf.csv"

shell:

"python3 scripts/bamToDF.py {params}"

## 8

#Perform the chimera search

csvDir=str(curDir + "scripts/bamToDf.csv")

rule chimericReads:

input:

csvFile="scripts/bamToDf.csv"

params:

refGenomeName=setRefGenomeName,

plasmidName=setPlasmidName,

prefix=csvDir

output:

"scripts/chimeraDf.csv"

shell:

"python3 scripts/210609_ChimeraSearch.py {params.plasmidName} {params.refGenomeName} {params.prefix}"

"""

#create a structural variations file.

rule svim_out:

input:

fa="ref/fullConstruct.fasta",

bam="alignment/merged_sorted.bam",

bai="alignment/merged_sorted.bam.bai"

output:

directory("svim_out")

shell:

"svim alignment {output} {input.bam} {input.fa}"

"""

**Supplement Code S2:** error analysis data extraction program for Nanopore data analysis

Code developed by S.M.Kashani and S.Hejazi

import csv

# This function reads a CSV file from the previous step and returns all rows except the first one as a list

def reader(file):

rows = []

f = open(file)

csv_reader = csv.reader(f, delimiter=',')

for row in csv_reader:

rows.append(row)

f.close()

del (rows[0]) # Remove the header row

return rows

#next function splits the cigar string into characters based onm the kind you selectiong that can be M for match, X for mismatch, D for deletion and I for insertion

def func(rows,i,cigar,kind):

num = [] # A list to store the numbers found in the cigar string

out = [] # A list to store the output data for each row

m = cigar.split(kind)

del(m[-1])

cnt = -1

out.append(rows[i][1]) # Add the sequence ID to the output list

for j in m:

# Loop through each character in the cigar string and extract the numbers

while True:

try:

int(j[cnt])

cnt -=1

except:

if len(j)>0:

num.append(int(j[cnt+1:-1]+j[-1]))

cnt =-1

break

num.sort(reverse=True)# Sort the list of numbers in descending order

SUM = sum(num) # Calculate the sum of all numbers in the list

out.append(SUM) # Add the sum to the output list

out.append(len(num)) # Add the number of elements in the num list to the output list

if len(num) != 0:

out.append(SUM/len(num)) # Calculate the average of the numbers in the num list and add it to the output list

else:

out.append("NA")

for i in num:

out.append(i)

return out

# This function generates a new CSV file that contains the analysis results

def CSV_GENERATOR(src,output_name,kind):

header = ["Query_name", "Length of SeQ", "Sum", "Number of "+kind, "AVG"]

output = open(output_name,"w+",newline="")

scv_writer = csv.writer(output)

rows = reader(src)

scv_writer.writerow(header)

for i in range(len(rows)):

scv_writer.writerow(func(rows,i,rows[i][8],kind))

output.close()

# Can select file name, analysis you want (=, M , X, I , D) in the last line

CSV_GENERATOR('bamToDf.csv',"Mlet.csv",'M')

**Supplement code S3** : a python program written to map the error’s location on reference sequence

Coded by S.M.Kashani

|  |  |
| --- | --- |
| from importlib.resources import path |  |
|  | import re |
|  | import csv |
|  | import numpy as np |
|  |  |
|  | REFRENCE_LENGTH = 999 |
|  |  |
|  | def reader(file): |
|  | f = open(file) |
|  | rows = csv.reader(f, delimiter=',') |
|  | rows = list(rows) |
|  | del (rows[0]) |
|  | return rows ### row[3] is start; row[8] is Cigar |
|  |  |
|  |  |
|  |  |
|  | def cigarToHeatMap(cigar, kind,start,lengthLimit): |
|  | start = int(start) |
|  | matches = re.findall(r"([0-9]+)([A-G,I-R, T-Z,=]+)", cigar) |
|  | # matches is a list of tuple and each tuple is like ('22','D')# |
|  |  |
|  | output = [0 for _ in range(lengthLimit)] |
|  | for match in matches: |
|  | if match[1] == kind: |
|  | for k in range(int(match[0])): |
|  | try: |
|  | output[start + k] = 1 |
|  | except: |
|  | break |
|  | start+= int(match[0]) |
|  | return output |
|  |  |
|  |  |
|  | def heatMapGenerator(kinds, row,lengthLimit): |
|  | hMap = {kind: None for kind in kinds} |
|  | for kind in kinds: |
|  | hMap[kind] = cigarToHeatMap(cigar = row[8], kind = kind, start = row[3],lengthLimit=lengthLimit) |
|  | hMap[kind] = hMap[kind] |
|  | return hMap |
|  |  |
|  | def conditionedHeatMapGenerator(path,condition = 700,lengthLimit = REFRENCE_LENGTH,kinds= ['X','I','D']): |
|  | hMaps = {kind: [0 for _ in range(lengthLimit)] for kind in kinds} |
|  | rows = reader(path) |
|  | x= 0 |
|  | for row in rows: |
|  | #progress keeper |
|  | x += 1 |
|  | if(x%1000==0): |
|  | print("progress: " + str(int(x/len(rows)*100))+'%') |
|  | #creat a dic of heatmaps for each kind |
|  | hMap = heatMapGenerator(kinds = kinds+['='], row = row ,lengthLimit = lengthLimit) |
|  | if sum(hMap['=']) + sum(hMap['X']) > condition: |
|  | for kind in kinds: |
|  | hMaps[kind] = np.add(hMaps[kind], hMap[kind]) |
|  | return hMaps |
|  |  |
|  | def CSVGenerator(dict,output_name,kinds): |
|  | output = open(output_name,"w+",newline="") |
|  | writer = csv.writer(output) |
|  | for kind in kinds: |
|  | dict[kind] = list(dict[kind]) |
|  | dict[kind].insert(0,kind) |
|  | writer.writerow(dict[kind]) |
|  | output.close() |
|  |  |

**Supplement code S4** : a python program written to fit Lorentzian function to emission data of sfGFP and measures FWHM of the peak.

Coded by Sepehr Hejazi

import pandas as pd

import numpy as np

import matplotlib.pyplot as plt

from scipy.optimize import curve_fit

# Load your Excel file

excel_file_path = 'complete all.xlsx'

df = pd.read_excel(excel_file_path)

# Assuming the first column contains wavelengths

wavelengths = df.iloc[:, 0]

# Select columns containing fluorescence intensities (starting from the 6th column)

intensity_columns = df.columns[3:4]

# Create an empty DataFrame to store fitted parameters for each sample

fit_results_df = pd.DataFrame(columns=['Sample', 'Amplitude', 'Center', 'FWHM'])

# Define a Lorentzian function for fitting

def lorentzian(x, amplitude, center, fwhm):

return (amplitude / np.pi) * (0.5 * fwhm) / ((x - center)**2 + (0.5 * fwhm)**2)

# Iterate through each sample column

for sample_column in intensity_columns:

# Extract fluorescence intensity for the current sample

intensity = df[sample_column]

# Normalize intensity to its maximum value in the column

intensity_normalized = intensity / intensity.max()

# Initial guess for the parameters

initial_amplitude_guess = 1

initial_center_guess = 508

initial_fwhm_guess = 49 # Rough estimate based on the data range

initial_guess = [initial_amplitude_guess, initial_center_guess, initial_fwhm_guess]

# Fit the Lorentzian curve to the data

params, _ = curve_fit(lorentzian, wavelengths, intensity_normalized, p0=initial_guess)

# Extract fitted parameters

amplitude, center, fwhm = params

# Store results in the DataFrame

fit_results_df = fit_results_df.append({

'Sample': sample_column,

'Amplitude': amplitude,

'Center': center,

'FWHM': fwhm

}, ignore_index=True)

# Generate fitted curve

fitted_curve = lorentzian(wavelengths, amplitude, center, fwhm)

# Plot the original data and the fitted Lorentzian curve

plt.plot(wavelengths, intensity_normalized, label=f'Normalized Fluorescence Intensity')

plt.plot(wavelengths, fitted_curve, label=f'Fitted Lorentzian Curve', linestyle='--')

plt.axhline(y=0.5, color='r', linestyle='--', label='Half-maximum Intensity')

plt.axvline(x=center, color='g', linestyle='--', label=f'Peak Wavelength (Fitted)')

# Customize the plot

#plt.title('Fitting Lorentzian Curve to Normalized Fluorescence Spectrum')

plt.xlabel('Wavelength (nm)')

plt.ylabel('Normalized Fluorescence Intensity')

# Adjust figure size and legend placement

plt.gcf().set_size_inches(10, 6) # Change the figure size as needed

plt.legend(bbox_to_anchor=(1.05, 1), loc='upper left', borderaxespad=0.)

# Save high-resolution image

plt.savefig('fitting_curve_high_res.png', dpi=300, bbox_inches='tight')

plt.show()

# Save fitted parameters to an Excel file

#fit_results_df.to_excel('fit_eppcr3.xlsx', index=False)

# Print the fitted parameters

print(fit_results_df)

## S8. length distribution of sequenced data

The following figures are histograms of length distribution of sequenced data. Linear templates from IDT were ordered (999bp) and amplified. Pjl1-sfGFP vector (2486 bp) is also amplified and sequenced.


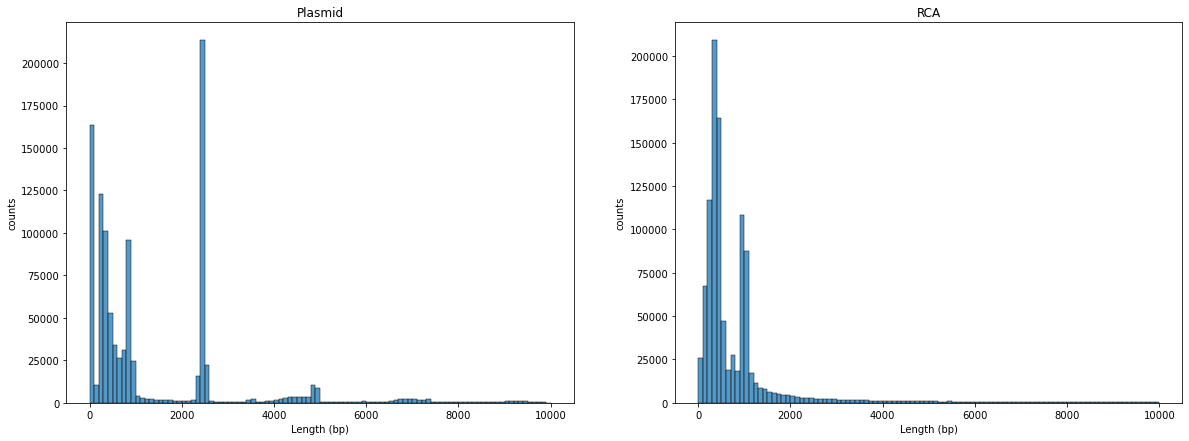


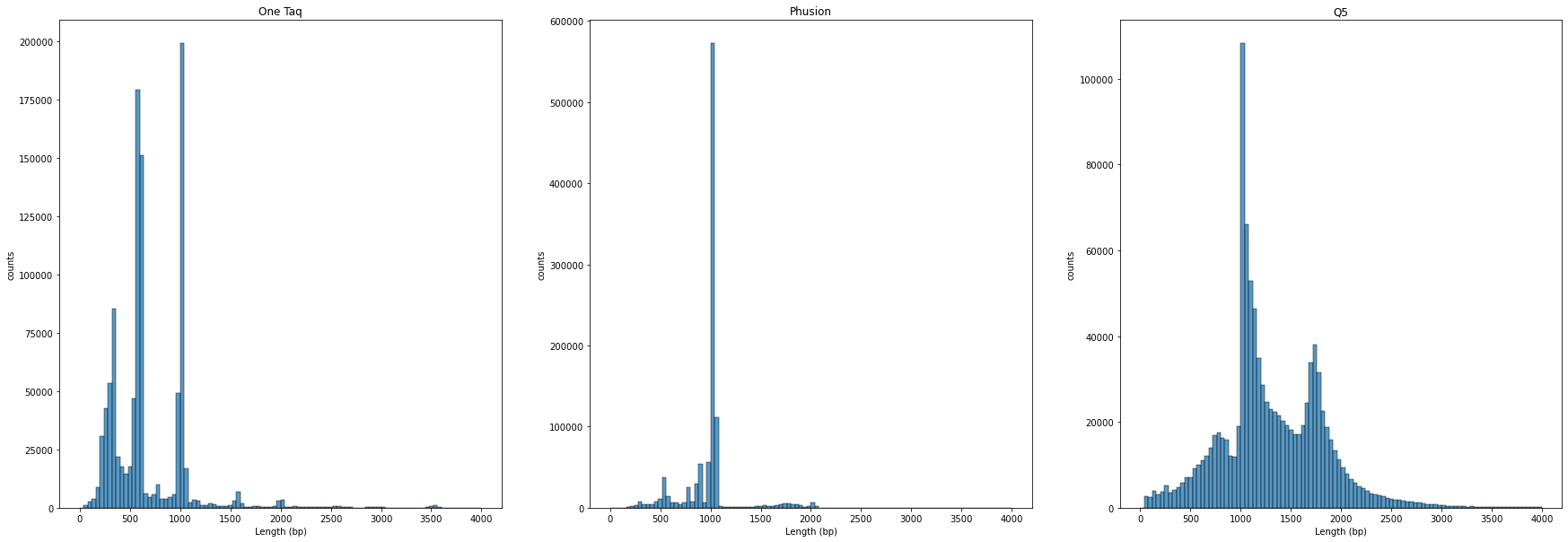
 **Figure S6** - Length distribution of ONT nanopore reads of different PCR amplification methods.

## References

Vilkhovoy M, Horvath N, Shih C-H, Wayman J, Calhoun K, Swartz J, Varner J. 2018. Sequence Specific Modeling of E. coli Cell-Free Protein Synthesis. *ACS Synth. Biol.*:139774.
